# Mapping evidence on management of cervical cancer in sub-Saharan Africa: scoping review protocol

**DOI:** 10.1186/s13643-021-01740-3

**Published:** 2021-06-21

**Authors:** Petmore Zibako, Mbuzeleni Hlongwa, Nomsa Tsikai, Sarah Manyame, Themba G. Ginindza

**Affiliations:** 1 Discipline of Public Health Medicine, School of Nursing and Public Health, University of KwaZulu-Natal, 2nd Floor George Campbell Building, Howard College Campus, Durban, 4041 South Africa; 2 College of Health Sciences, University of Zimbabwe, Harare, MP167 Zimbabwe

**Keywords:** Cervical cancer management, Control, prevention, Screening, Diagnosis, HPV vaccine, Treatment, Chemotherapy, Radiotherapy, Sub-Saharan Africa

## Abstract

**Background:**

Cancer is a non-communicable disease and is the number 2 leading cause of death globally. Among all cancers, cervical cancer is the number 1 killer of women in low-income countries (LICs). Cervical cancer is a well understood preventable cancer. The rates of cervical cancer are very varied and inversely proportional to the effectiveness of disease management policies. Management of cervical cancer includes prevention, screening, diagnosis and treatment. The main objective of this scoping review is to map the evidence on cervical cancer management in sub-Saharan Africa (SSA) to understand the coverage of cervical cancer prevention and treatment services and provide an opportunity to generate knowledge on the risk factors, attitudes and practices extendable globally.

**Methods and analysis:**

This review will be guided by Arksey and O’Malley’s framework recommended for conducting scoping review studies. The Preferred Reporting Items for Systematic Review and Meta-Analysis extension for Scoping Reviews (PRISMA-Scr) checklist will also be completed to ensure that the review adheres to the sound methodological rigour acceptable for scoping review studies. The following electronic databases will be searched for potentially eligible articles: PubMed, Ebsco Host, Scopus and Cochrane Database of Systematic Reviews. Study screening procedures recommended by Higgins and Deeks will be followed. A narrative synthesis will be used, with data synthesised and interpreted using sifting, charting and sorting based on themes and key issues.

**Discussion:**

Cervical cancer can become a disease of the past with a proper control strategy in place. It is therefore imperative to map available evidence on the management of cervical cancer to inform policy and advocacy action. More knowledge on the status quo will guide policymakers in ensuring cancer management guiding policies are formulated/updated/revised accordingly.

**Systematic review registration:**

Not registered with PROSPERO (not needed).

**Protocol and registration:**

This scoping review was not registered.

## Background

Cancer of the cervix is caused by persistent infection with high-risk types of human papillomavirus (HPV) [1]. Persistent HPV infection causes inactivation of pRb and p53 tumour suppression genes by E6 and E7 proteins of the HPV genome leading to cervical intraepithelial neoplasia (CIN) which eventually develop into cancer [1]. There are more than one hundred types but approximately 13 genotypes of HPV can cause cervical cancer (CC) [2], and HPV 16 and 18 account for 70% of all cancer of the cervix [3]. HPV is sexually transmitted, and high-risk HPV genotypes are often present in 99.7% of CC specimens [4].

CC is a preventable and treatable disease [5]. HPV can persist and cause pre-cancerous changes in cells which are called CIN [6]. The development from low-grade CIN to invasive CC is approximately 10 to 20 years and 5 to 10 years in people with immune suppression like those with HIV infection [7–10]. Screening for CIN and early treatment to remove pre-cancerous changes is effective in preventing invasive CC [11]. Methods of identifying CIN include the Papanicolaou (Pap) test, visual inspection with acetic acid (VIAC) and HPV DNA test [11].

Approximately 570,000 new CC cases and 311,000 deaths were recorded worldwide in 2018, and globally, CC is at number 4 of all cancers [6]. In sub-Saharan Africa (SSA), CC is the leading cause of cancer deaths among females [12]. It is estimated that 90% of CC deaths occurred in developing countries, 25% in India (67,500), 60,100 deaths in Africa, 144,400 in Asia and 28,600 in the Caribbean Bay and Latin America [13]. Variations in CC rates are due to the difference in availability of screening that provides for the detection and treatment of precancerous lesions as well as HPV infection prevalence [14]. HPV infection prevalence is highest in Africa (21%), Latin America and the Caribbean (16%), Asia (9%) and North America (5%) [13].

In some Western countries, CC rates decreased by 65% [13]. In Norway, CC incidence decreased from 18.7 per 100,000 in 1970 to 9.6 per 100,000 in 2011 due to the well-established screening programmes [13]. In the USA, the overall cancer death rate dropped by 27% from 1991 to 2016 while in developing countries, mortality rates were 2-fold higher for CC over the same period [15]. In the USA, CC was the leading cause of cancer death among women in 1930; the death rate was 36/100,000, dropped to 5.6 in 1975 and to 2.3 in 2015 due to the development and implementation of the PAP test, improved treatment of CC and HPV vaccination with an uptake rate of 47.5% in 2016 [16]. In SSA, the greatest threat to CC management is the unavailability of vaccines, information, treatment and monitoring. CC is a neglected area of women’s health in LICs [17].

LICs recognise the importance of HPV vaccination, but getting the resources for the vaccine has been a challenge. Gardasil4 and Cervarix vaccines are available for protection against two types of HPV that cause approximately 70% of CC cases [13]. Gardasil4 and Cervarix are 90% effective in preventing HPV 16/18; Cervarix is 70% effective against HPV 31/45 infections [18]. The third vaccine, Gardasil9, protects against the following HPV genotypes: 6, 11, 16, 18, 31, 33, 45, 52, and 58 [19]. Gardasil4 and Gardasil9 prevent anogenital warts that are caused by HPV 6 and 11 [19]. WHO recommends two doses for 9- to 14-year-old girls and three doses for those above 15 years and the immune-compromised people [19]. The major barrier to vaccination is the associated high cost [20]. This research is there to find the most cost-effective way from literature as well as other factors that can lead to high HPV coverage. Of 20 countries with the highest incidence of CC, 16 are in Africa [21], and the incidence rates in SSA are above 40 per 100,000 women [22]. There is a need that the prevention method of HPV vaccination outcomes is improved mainly through high coverage.

On the other hand, HPV vaccines do not protect against already existing infections and do not protect against all the types of HPV that cause CC [23]; therefore, screening is a critical component of CC management. There is a need to enhance the uptake of screening by finding out the barriers to screening service uptake to inform on national screening programmes and policy formulation. The Papanicolaou test is the most common screening tool for CC in developed countries, while in LICs, it is generally inaccessible or met with resistance due to misinformation or poor attitudes [24]. Cytology screening methods like the Papanicolaou test have limited relevance in LICs because of limited infrastructure and trained personnel like cyto-technicians [25]. It is imperative to note here that a single round of HPV testing can reduce the number of CC deaths by about 50% [13], but costs, infrastructure and specificity issues limit its use in LICs [25]. Visual inspection with acetic acid (VIAC) is the common screening method used in LICs which provide high screening coverage because it is a simple and inexpensive test, despite its drawbacks of variability and subjectivity of results interpretation which can result in false positives and overtreatment [25].

CC is the most screened cancer worldwide [26]. Cervical cytology screening programmes using Papanicolaou every 3 to 4 years reduced CC incidence and mortality by approximately 80% in developed countries like North America, Europe, New Zealand, Japan and Australia [27]. Screening tests like liquid-based cytology, conventional cytology, HPV testing, visual inspection with acetic acid (VIAc) and visual inspection with Lugol’s iodine (VILI) can detect CIN, if done with quality assurance [6]. The mechanisms of screening are already established scientifically what is needed is to make sure that the screening services are provided and utilised hence the need to find out barriers to screening services uptake with the intention to come up with solutions that can be recommended to improve screening services uptake in order to reduce mortality from CC. Coverage of CC screening in LMICs is an averagely 19% [28]. Screening all women in a targeted age group every 3 years can prevent 91% of CC cases [29].

Incidence, survival and death rates can be used to measure progress in CC control with death rates being the best indicator [6]. Approximately, 90% of CC deaths occur in LICs [30], but it is technically possible to control CC mortality globally. In Zimbabwe, Gambia and Uganda, a 5-year age-adjusted standardised survival was as low as 19%, 22% and 13%, respectively [31]. Age-standardised death rate per 100,000 women in east Africa was 12 times as high as in Western Europe (25.3% versus 2%) [14]. Women in developed countries have a 208% greater chance of being successfully treated compared with women in LICs [32]. There is a need to keep checking the status of radiotherapy and chemotherapy so as to keep improving the standard of care so as to increase survival rate and successfully treated patients.

The 5-year survival rate is 91% for localised CC at diagnosis which falls to 57% for distant stage CC [33]. Stages of advanced CC are IB, IIA, IIIA, IIIB, IIIC, IVA and IVB, and they require standard curative treatment with external beam radiation, brachytherapy with or without chemotherapy [34]. There is a need to know the reasons why CC patients presents often with late-stage CC at diagnosis so as to improve survival rates as well as general treatment outcomes like quality of life. The efficacy of CC treatment depends on the stage of cancer at diagnosis [35]. Approximately 80% of patients present with advanced stage of CC at diagnosis in LICs [36].

HIV-positive people are a high-risk group for HPV infections [37], yet SSA accounts for over 70% of the global HIV/AIDS burden [38]. HIV-positive women have a 6-fold excess risk of developing CC because of immune-suppression [39]. Almost 6% of women with CC are HIV positive, and about 5% of all CC cases are due to HIV infection [6]. It is important to keep checking from empirical evidence how HIV-positive CC patients are copying with radiotherapy and chemotherapy.

Cervical cancer control is defined as activities to reduce the CC burden through dissemination and implementation of evidence-based interventions [40]. The interventions include prevention, early detection (screening and diagnosis) and treatment [40]. Improvements in CC control involve addressing system challenges and changing policies of public health structural interventions which alter the structural context for health and are often politicised [41]. Prevention of suffering and death from CC is a human rights issue hence the need to continuously update and collate available evidence on CC that can improve CC management. This study will help as a stepping stone to achieve WHO’s strategy to eliminate CC as a public health problem: elimination level of 4/100,000 women cases, HPV vaccination coverage to 90%, twice-lifetime CC screening to 70% and treatment of pre-invasive lesions to 90% [42].

The proposed hypothesis for this review is CC management needs improvement in SSA. The main research question is: What evidence is there on CC management in SSA? The sub-research questions include the following:

- What are the factors associated with high HPV vaccine coverage in SSA?
- What are the barriers to CC screening uptake in SSA?
- What factors are associated with late-stage CC presentation at diagnosis in SSA?
- What is the status of radiotherapy and chemotherapy in CC management in SSA?

The aim of this review is to map evidence on CC management in SSA. The objectives of the study are as follows:

- To explore the factors that are associated with high HPV vaccine coverage in SSA
- To determine the barriers to CC screening uptake in SSA
- To find out the factors that are associated with late-stage CC presentation at diagnosis in SSA
- To establish the status of radiotherapy and chemotherapy in CC management in SSA

## Methods

### Scoping review

The proposed scoping review will be conducted in accordance with Arksey and O’Malley’s (2005) scoping review framework. Arksey and O’Malley [43] indicate that the following steps should be undertaken when conducting scoping reviews: (a) identifying the research question, (b) identifying relevant studies, (c) selecting studies, (d) charting the data and (e) collating, summarising and reporting the results. The review will also follow the steps and guidelines outlined in the PRISMA-Extension for Scoping Reviews (PRISMA-ScR) checklist [44].

### Identifying relevant studies

An initial search was performed to determine whether a previous review addressing this topic in SSA was conducted or was in progress. There were no complete or in-process reviews focusing on the coverage of cervical cancer prevention and treatment services in SSA. Based on the review question, the search strategy was developed by identifying the key concepts using the PICO (Problem/Intervention/Comparison/Outcome) approach [45] and further developing the search strategy using controlled vocabulary such as MeSH (Medical Subject Headings) terms. Papers published on CC management will be reviewed for each of the following topics: CC prevention, detection (screening and diagnosis) and treatment. A healthcare librarian from the University of Zimbabwe was consulted for the search strategy of electronic databases. The following key search words will be used: cervical cancer management or cervical cancer control, cervical cancer screening or VIAC or Pap smear or HPV testing, cervical cancer prevention or HPV vaccine, cervical cancer treatment or cervical cancer chemotherapy, and cervical cancer radiotherapy. African country names and truncated terms such as ‘east* Africa’ will also be used to ensure that articles indexed using African country-specific names or regional terms are retrieved. The operator ‘or’ will be used to combine synonyms and the operator ‘and’ to filter the results which contain all the required terms. The Peer Review of Electronic Search Strategies (PRESS) Checklist is used for the search strategy. The databases to be searched include PubMed, Ebsco Host, Scopus and Cochrane Database of Systematic Review.

### Study selection

**Fig. 1:**
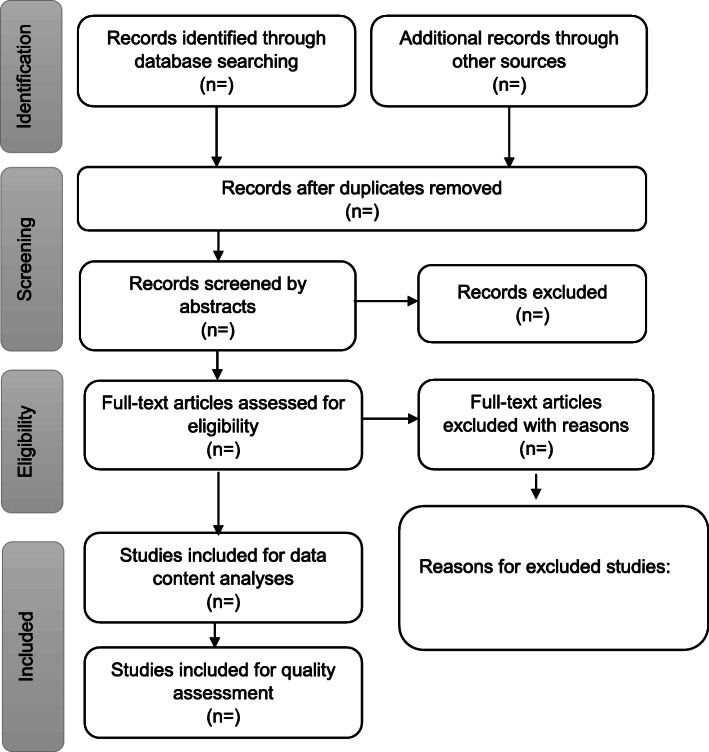
PRISMA flow diagram of the study selection process

Two independent reviewers will conduct the abstract and full article screening. The literature will include published peer-reviewed journal articles with evidence of empirical design utilising either qualitative, quantitative or mixed method research approach addressing the research questions. The screening procedure will be guided by Higgins and Deeks’ framework [46]. All articles identified to be potentially eligible for inclusion in this review will be obtained in full texts. These articles will be then be exported to reference management software, EndNote version X7. Duplicates will then be removed before further screenings (abstract and full article) are conducted. The PRISMA (Preferred Reporting Items for Systematic Review and Meta-Analysis) flow chart will be used to display the screening results of studies [47] (Fig. 1).

### Eligibility criteria

#### Inclusion criteria

The inclusion criteria were guided by the following principles to determine the articles relevant for this review:

- Studies presenting evidence on cervical cancer.
- Studies presenting evidence conducted in SSA.
- No limits will be applied for the publication date of included studies.
- All study designs will be considered.

#### Exclusion criteria

Studies that do not focus on humans, as well as those written in languages other than English, will be excluded. Non-empirical material like book chapters, opinion papers, commentaries and editorials will also not be included.

### Data extraction and charting

A data collection instrument (Table 1) was developed to confirm the study characteristics as well as relevance. Data will be extracted by the principal investigator. The data extraction form will include the following elements: author(s), year of publication, title of study, country, study aim(s) or research question, study design, study setting (urban/rural), study population, sample size, key findings that relate to the review question, study limitations and implications, and interpretations and conclusions from the authors. Data will be entered into Access, and qualitative data will be uploaded in NVivo, a computer-assisted qualitative data analysis software.

**Table 1:** Data extraction form

Area of interest (broad category)	Author and publication year	Title	Aims or research questions	Study design	Country	Study population	Sample size	Key findings	Other findings	Study limitations	Conclusions	Additional comments
Prevention												
Diagnosis												
Barriers of Screening Uptake/uptake rate												
Surgery												
Chemotherapy												
Radiotherapy												

### Data analysis

A narrative synthesis will be used, with data synthesised and interpreted using sifting, charting and sorting based on themes, key issues and type of study. Citation tracking will be done using the Reference Manager Software in Endnote version X7. Data analysis and tabulation of the findings will be done using Review Manager (RevMan) [48]. The narrative synthesis approach [49] will help summarise and identify the patterns across studies using tabulations, clustering, textual descriptions, conceptual triangulation (concept mapping) and thematic analysis. Textual data summary will be tabulated from qualitative, mixed methods and quantitative studies. Descriptive statistics will also be used to quantify studies based on the patterns identified. Directed content analysis methods will be used on abstracted data to identify patterns or themes that characterise factors that affect CC management.

### Quality control and assessment

Studies that will be published between the research and report writing will be obtained by subscribing to updates to databases using the search domains used during the literature search. Data will be extracted by the principal investigator, and accuracy will be checked by a second reviewer. Studies with uncertainties about their inclusion will be discussed with a third reviewer.

The quality of evidence will be assessed based on guidance in the National Institute for Health and Care Excellence single technology appraisal Specification for Manufacturer/Sponsor Submission of Evidence adapted from the Centre for Reviews and Dissemination’s guidance for undertaking reviews in healthcare [49].

Mixed Method Quality Appraisal Tool will be used for quality assurance [27], with aspects like sampling frame, stating hypothesis, defined target population, and defined study population, stated study setting, dates study was conducted, eligibility criteria, selection into the study, justification of number of participants, stated number of participants at the beginning of the study, methods of data collection, reliability/repeatability measurement, methods of follow up, were participants at each stage specified, were the reasons for loss to follow up quantified, was missing data accounted for in the analysis, was the impact of bias estimated quantitatively, were used to assess the quality of included studies. The studies will be rated as good quality, fair quality or poor quality with comments on each study. Dissemination of the results will include publications in journals and presentations at health conferences.

## Discussion

CC deaths and incidence rates are still high in SSA [50]. The rate of control achieved depends on prevention (vaccination and screening) policies. Vaccination of girls has a long term effect on cancer rates because of the long period (10 to 20) [30] from HPV infection to invasive CC; hence, control will remain crucial for a long time into the future.

Global research has shown that among the ways to prevent CC, CC screening and follow-up has the greatest or second greatest impact after vaccination; hence, lives can be saved by the comprehensive application of available evidence-based interventions to all females who could be affected by CC. The results of the scoping review will be used to inform health policy and knowledge to end-users regarding strategies that can be used to facilitate CC control. The review will be used to identify research gaps that need to be addressed in CC management. Furthermore, this review will provide a complete and reliable picture of how CC control is being managed in the region, the challenges and opportunities of CC management will be highlighted [51]. The results of this review may likely contribute to women’s understanding of the relationship between HPV and CC for them to make appropriate, evidence-based decisions on available prevention strategies [52]. This scoping review will contribute to informing guidelines regarding CC control and management.

The outlook for CC control is bright since the knowledge of what to do is there and the tools to do prophylactic interventions which include, vaccination of the girls, screening and preventive treatment for adult women are well known. Treatment of precancerous lesions is of low cost compared to the cost of invasive cervical cancer treatment (which is not readily accessible to many women) [53]. It is detrimental to fail to use tools and knowledge for HPV vaccination, screening and preventive treatment that have saved lives in HICs. Communities need to act urgently to save women and girls from this monster called CC. CC is a well-known and preventable cancer, and the rates of CC are very varied and are inversely proportional to the effectiveness of prevention policies. We could not find mapped evidence on CC management processes to explain the increasing CC deaths and incidence.

## Data Availability

All data generated or analysed during this study will be included in the published systematic review article.

## Abbreviations

CC: Cervical cancer
CIN: Cervical intraepithelial neoplasia
HICs: High-income countries
HPV: Human papilloma virus
LICs: Low-income countries
LMICs: Low- and middle-income countries
MeSH: Medical Subject Headings
PAP: Papanicolaou
PICO: Problem/Intervention/Comparison/Outcome
PRESS: Peer Review of Electronic Search Strategies
PRISMA: Preferred Reporting Items for Systematic Review and Meta-Analysis
PROSPERO: International prospective register of systematic reviews
RevMan: Review Manager
SSA: Sub-Saharan Africa
VIAC: Visual Inspection with Acetic Acid and Camera
